# Organizational mechanisms in digital public health transformation: change communication, collective sensemaking, and cross-functional collaboration

**DOI:** 10.3389/fpubh.2026.1978109

**Published:** 2026-10-30

**Authors:** Linxuan Ying

**Affiliations:** Research School of Management, ANU College of Business and Economics, The Australian National University, Canberra, ACT, Australia

**Keywords:** change communication, collective sensemaking, cross-functional collaboration, digital public health, digital transformation

## Abstract

**Introduction:**

Digital public health transformation extends beyond technology deployment and requires organizational processes that align interpretation, coordination, governance, and population-health goals. Existing research examines change communication, collective sensemaking, and cross-functional collaboration separately, with limited integration of their boundaries, recursive interactions, cross-level transmission, and multilevel outcomes. This review develops an organizational explanation of how these mechanisms may support transformation.

**Methods:**

A narrative integrative review synthesized theoretical, empirical, policy, and technical literature from organizational science, implementation science, digital public health, data governance, and artificial intelligence governance. Searches covered PubMed/MEDLINE, Embase, Scopus, Web of Science Core Collection, APA PsycINFO, CINAHL, IEEE Xplore, ProQuest Public Health Database, Business Source Complete, and traceable institutional sources from inception to October 1, 2026. Sources were purposively selected for relevance, contribution, public health applicability, analytical level, and traceability. Synthesis used construct coding, within-theme comparison, framework comparison, cross-level mechanism mapping, and analysis of competing explanations and evidence boundaries.

**Results:**

Change communication establishes credible and contestable direction; collective sensemaking develops shared problem representations; cross-functional collaboration translates shared understanding into coordinated action. They operate recursively rather than sequentially. A seven-layer framework links structural contextual conditions, organizational mechanisms, potential mediating capabilities, implementation outcomes, organizational or system outcomes, population-level service and equity outcomes, and population health outcomes. Team psychological safety moderates the relationship between proximal support conditions and risk-detection behaviors, which feed back into the mechanisms. Organizational readiness for change, knowledge absorption and translation, interoperability governance, organizational learning, and resilience are proposed as mediating capabilities. Power and equity governance, community decision rights, representativeness, vendor accountability, auditing, appeals, portability, and exit management cut across the framework. The model complements CFIR, NASSS, i-PARIHS, EPIS, and sociotechnical approaches by specifying observable interactive mechanisms and feedback pathways.

**Discussion:**

The framework provides a testable explanatory structure and governance-diagnostic tool for surveillance, data infrastructures, laboratory networks, artificial intelligence, and community co-production. Evidence more strongly supports organizational and system processes than causal effects on population health. Longitudinal, multilevel, and mechanism-oriented studies should test mediation, moderation, feedback, equity, and cross-level transmission.

## 1 Introduction

Digital public health can be differentiated into three levels: digitization of information, digitalization of processes, and reconfiguration of organizational operating models; organizational culture, governance, population-health goals, and cross-sector practice cut across all three (1, 2). Real-time data, mobile technologies, network science, and public participation can expand surveillance and intervention capacity, but they also bring privacy, validation of effectiveness, scale-up, data rights, and intergenerational equity into the public governance agenda (3, 4). Accordingly, deployment of digital tools is a condition for transformation rather than a sufficient explanation of organizational adoption, sustained use, or population-health value.

Digital transformation changes organizational pathways of value creation, structures, legitimacy, professional boundaries, and interorganizational relationships. Organizational inertia, skill gaps, institutional constraints, user participation, and governance coordination jointly shape transformation trajectories (5–8). The competencies required of the public health workforce extend beyond software operation to encompass data science, informatics, ethics, communication, implementation, interdisciplinary collaboration, and leadership (9–11). Experience from public health emergencies further suggests that whether digital surveillance, contact tracing, and modeling become usable capabilities may depend jointly on data quality, public acceptability, and institutional coordination (12).

Existing literature addresses communication, sensemaking, team coordination, implementation context, and digital governance separately, but provides no consistent account of mechanism boundaries, cross-level transmission, or links to population outcomes. This review focuses on three interdependent questions: how organizations interpret the need for transformation; how heterogeneous professions and communities develop shared problem representations; and how multiple parties translate shared understanding into sustained joint action. The primary readership comprises digital public health researchers, public health agency managers, data and algorithm governance personnel, and interorganizational implementation teams. The relevant constructs are operationalized in the contexts of surveillance, data infrastructures, laboratory networks, artificial intelligence (AI), and community co-production.

## 2 Review methods and analytical approach

### 2.1 Review design and data sources

This review adopted a narrative integrative design to synthesize theoretical and empirical evidence from organizational science, implementation science, digital public health, data governance, and artificial intelligence (AI) governance, and to compare construct boundaries, mechanism relationships, and conditions of applicability. Narrative integration does not pool effect sizes; instead, it emphasizes interdisciplinary conceptual comparison and integration of theoretical pathways. Sources included PubMed/MEDLINE (Medical Literature Analysis and Retrieval System Online), Embase, Scopus, Web of Science Core Collection, American Psychological Association (APA) PsycINFO, Cumulative Index to Nursing and Allied Health Literature (CINAHL), Institute of Electrical and Electronics Engineers (IEEE) Xplore, ProQuest Public Health Database, and Business Source Complete. Policy and technical materials were obtained from the World Health Organization, the Centers for Disease Control and Prevention, the National Institute of Standards and Technology, European Union regulatory platforms, and other traceable public health institutions. The search was completed on October 1, 2026, and covered each database from inception to that date. Institutional materials were included only when the issuing body and document version or publication date were clearly specified and verifiable.

### 2.2 Search strategy and selection principles

The search combined concepts relating to digital public health and digital transformation; organizational change and communication; collective sensemaking; cross-functional and interorganizational collaboration; implementation frameworks; data and algorithm governance; power and equity; and population health outcomes. In PubMed, for example, the search strategy was: (“Public Health Informatics” OR “Public Health” OR “Digital Technology” OR “Artificial Intelligence”) AND (“Organizational Innovation” OR “Communication” OR “Interinstitutional Relations” OR “Interdisciplinary Communication” OR “Cooperative Behavior”) AND (“Organizational Culture” OR “Decision Making, Organizational” OR “Health Equity”). Title- and abstract-based terms additionally included “digital public health,” “digital transformation,” “change communication,” “sensemaking,” “cross-functional collaboration,” “implementation framework,” “algorithmic governance,” “generative artificial intelligence,” “foundation model,” and “vendor governance.” Equivalent adaptations were made for database-specific fields, controlled vocabulary, and syntax. Reference-list screening and forward citation tracking were used to identify foundational theories, major frameworks, recent public health studies, and governance documents. Sources were selected on the basis of topical relevance, theoretical or empirical contribution, direct relevance to public health contexts, alignment of the level of analysis, and source traceability.

### 2.3 Data extraction and narrative synthesis

Records were first screened by title and abstract, followed by full-text assessment of their theoretical or empirical contribution, applicability to public health, level of analysis, and source traceability. Eligible materials included theoretical studies, empirical studies, reviews, and policy or technical documents with clearly identified issuing organizations and version information. Materials were excluded if they reported technical performance without addressing organizational or governance processes, focused solely on individual clinical treatment without relevance to public health systems, could not be traced to a source, were duplicates, or were unrelated to the review questions. One researcher conducted source identification, full-text assessment, extraction, and initial coding; a second researcher reviewed borderline inclusion decisions, construct coding, and citation information. Disagreements were resolved by checking the original source and reapplying the prespecified criteria. Extracted information included study context, level of analysis, theoretical framework, construct definitions, contextual conditions, mechanisms or capabilities, implementation and system outcomes, population outcomes, principal findings, and limitations. Synthesis involved coding of conceptual boundaries, within-theme comparison, framework comparison, cross-level mechanism mapping, and analysis of competing explanations and evidence boundaries. Peer-reviewed studies were used to support empirical judgments, whereas policy and technical documents were used to describe normative and governance requirements rather than evidence of intervention effects. Article titles, authors, journals or issuing bodies, year, volume, issue, page range or article number, digital object identifier (DOI), and document version were verified against publisher or official sources. Given the heterogeneity of study designs, contexts, and outcomes, effect sizes were not pooled.

## 3 Evidence synthesis and thematic analysis

### 3.1 Organizational characteristics and analytical boundaries of digital public health transformation

#### 3.1.1 From technology deployment to sociotechnical system reconfiguration

Digital public health systems comprise technologies, people, workflows, organizational rules, and external institutions. Sociotechnical evidence indicates that hardware and software, content, human-computer interfaces, people, communication processes, organizational policies, external regulation, and monitoring feedback remain continuously coupled; optimization of a single element may be offset by weaknesses elsewhere (13). Professional identities, power relations, redistribution of work, and established norms can alter the adoption trajectory of the same technology across organizations (14). Technological complexity, the value proposition, adopter systems, organizational capacity, and the external environment jointly shape nonadoption, abandonment, scale-up, spread, and sustainability (15). Because technology is shaped by practice and can in turn reinforce or alter organizational structures, digital public health transformation should be understood as ongoing sociotechnical reconfiguration rather than a one-time installation (16).

#### 3.1.2 Structural complexity of public health contexts

Digital public health is more boundary-spanning than implementation of an information system within a single organization. Data may originate from healthcare institutions, laboratories, community organizations, schools, environmental monitoring systems, mobile devices, and commercial platforms. Decision-makers are distributed across local, regional, national, and international levels. Beneficiaries include both current service users and at-risk populations not yet reached by services. Five interrelated challenges follow: governance objectives such as efficiency, early warning, privacy protection, equity governance, and maintenance of public trust may compete; different professions and community knowledge systems apply different standards of evidence; authority over data control, algorithmic accountability, and resource allocation is dispersed; emergency response, procurement, deliberation, and legislation operate on different timescales; and local adoption does not necessarily improve population coverage or equity.

Pre-existing power distributions, initial representativeness, and initial vendor dependence are structural contextual conditions that define the initial constraints and opportunities for the three organizational mechanisms. Authorization for participation, community decision rights, maintenance of representativeness, resource compensation, vendor accountability, independent auditing, appeals, portability, and exit management are governance practices that extend across the implementation life cycle. Analysis should specify who can define the problem, select data, approve models, interpret results, allocate resources, and decide whether to update or discontinue a system. It should also document the actual decision-making authority of minority groups, vendors’ effective control over data, interfaces, and model updates, and the capacity of public institutions to conduct independent audits, handle appeals, and exit in an orderly manner.

#### 3.1.3 Conceptual boundaries and construct roles

Change communication is an interactive process through which a shared interpretation is developed around the need for transformation, its direction and consequences, and the space for contestation. Collective sensemaking is an interpretive process through which actors jointly extract cues, negotiate identities, translate professional languages, and form shared problem representations. Cross-functional collaboration is an action process through which interdependent tasks are accomplished by complementary roles, knowledge integration, joint decision-making, and shared accountability. Public value goals, institutional complexity, resources, digital maturity, regulation, pre-existing power distributions, initial representativeness, and initial vendor dependence are antecedent or structural contextual conditions. Leader inclusiveness, reporting protection, and response mechanisms are team-level proximal support conditions. Authorization for participation, community decision rights, maintenance of representativeness, resource compensation, vendor accountability, independent auditing, appeals, portability, and exit management are cross-cutting governance practices. Team psychological safety is positioned as a team-level proximal moderator that helps explain whether support conditions are translated into anomaly reporting, voice, help-seeking, and assumption challenging. These behaviors are proximal behavioral outcomes and feedback signals rather than indicators of psychological safety. Internal organizational trust is a contextual perception at the individual or team level; interorganizational trust is a network-governance condition; and public trust is a social outcome at the organizational or system level. The actual level of measurement may vary by study design, and cross-level aggregation must meet the relevant statistical conditions. Organizational readiness for change, knowledge absorption and translation, interoperability governance, organizational learning, and resilience are distinct potential mediating capabilities. Adoption quality, workflow integration, timeliness of decision-making, and institutionalization are implementation outcomes. Data quality, latency, interorganizational coordination, governance accountability, and public trust are organizational or system outcomes. Coverage, service accessibility, subgroup performance, burden distribution, and equity gaps are population-level service and equity outcomes. Avoidable morbidity, mortality, functional loss, and health gains are population health outcomes (Table 1).

**Table 1:** Construct roles of organizational mechanisms, contextual conditions, mediating capabilities, and multilevel outcomes.

Analytical position/Construct	Operational definition	Construct role	Primary level(s)	Example evaluation indicators
Public value goals; institutional and network complexity; resources; digital maturity; regulation; pre-existing power distribution; initial representativeness; initial vendor dependence	Define the constraints, opportunities, and initial power conditions under which mechanisms operate	Antecedent/structural contextual conditions	Organization, network, community	Resource slack; network size; technological maturity; initial representativeness; initial vendor dependence
Leader inclusiveness, reporting protection, and response mechanisms	Provide proximal support for raising objections, reporting anomalies, and receiving feedback	Team-level proximal support conditions	Team, organization	Leader responsiveness; reporting protection; closed feedback loops
Authorization for participation; community decision rights; maintenance of representativeness; resource compensation; vendor accountability; independent auditing; appeals; portability; exit management	Define who can participate in decisions, how vendors and risks are overseen, and how errors are corrected or exit is managed	Cross-cutting governance practices	Organization, network, community	Actual decision-making authority; representativeness; compensation; vendor accountability; audit coverage; handling of appeals; portability; execution of exit
Change communication	Develop shared interpretations of necessity, direction, consequences, and the space for contestation	Component of the organizational mechanisms	Individual, team, organization	Goal comprehension; information credibility; responsiveness to feedback; access to feedback and voice channels
Collective sensemaking	Jointly extract cues, negotiate identities, translate language, and form shared problem representations	Component of the organizational mechanisms	Team, organization, network	Visibility of alternative explanations; records of dissent; shared representations; frequency of reassessment
Team psychological safety	Shared perception that reporting problems, challenging assumptions, and requesting help are safe	Team-level proximal moderating condition	Team	Safety of speaking up; tolerance of error; perceived nonpunitive reporting; safety of help-seeking; scale scores
Cross-functional collaboration	Complete interdependent tasks through role complementarity, knowledge integration, joint decision-making, and shared accountability	Component of the organizational mechanisms	Team, organization, network	Knowledge integration; clarity of responsibility; conflict repair; joint decision-making
Risk-detection behaviors	Behaviors such as anomaly reporting, voice, help-seeking, and assumption challenging that introduce problem cues into organizational processes	Proximal behavioral outcomes and feedback signals	Individual, team	Rate of anomaly reporting; voice; help-seeking; records of dissent; issue escalation
Organizational readiness for change	Members’ shared judgment of collective commitment and collective implementation efficacy	Potential mediating capability	Team, organization	Collective commitment; collective efficacy; intention to act
Knowledge absorption and translation	Capability to identify, absorb, translate, and apply external or cross-professional knowledge	Potential mediating capability	Team, organization, network	Knowledge acquisition; boundary-spanning translation; knowledge application; workflow adaptation
Interoperability governance	Create enforceable agreements on data definitions, standards, authorization, interfaces, and responsibilities	Potential mediating capability	Organization, network, system	Semantic comparability; interface consistency; clarity of authorization; responsibility tracking
Organizational learning	Translate anomalies, feedback, and performance information into updates of rules and practices	Potential mediating capability	Team, organization, network	Speed of correction; rule updates; implementation of after-action reviews; closed learning loops
Resilience	Maintain, restore, and adapt core public health functions under shocks and failures	Potential mediating capability	Organization, network, system	Continuity of core functions; recovery time; alternative pathways; adaptive capacity
Adoption quality, workflow integration, timeliness of decision-making, and institutionalization	Assess whether implementation is acceptable, feasible, contextually appropriate, and embedded in routine work	Implementation outcomes	Organization, network	Sustained use; time from signal to decision; workflow embedding; institutionalization
Data quality, latency, interorganizational coordination, governance accountability, and public trust	Assess whether the system produces interpretable and accountable public health operations	Organizational/system outcomes	Network, system	Completeness; latency; timeliness of coordination; auditing; public trust
Coverage, service accessibility, subgroup performance, burden distribution, and equity gaps	Assess whether different populations receive accessible and effective public health services with a reasonable distribution of burden	Population service and equity outcomes	Community, population	Coverage gaps; service use; subgroup performance; burden distribution
Morbidity, mortality, functional loss, and health gains	Assess whether system changes translate into observable changes in population health	Population health outcomes	Population	Morbidity; mortality; avoidable health loss; health gains

The five mediating capabilities are parallel constructs. Team psychological safety moderates the relationship between proximal support conditions and risk-detection behaviors. Anomaly reporting, voice, help-seeking, and assumption challenging are proximal behavioral outcomes and feedback signals. Public trust is a social outcome at the organizational or system level; it may be measured at the individual, community, or population level, but cross-level aggregation requires sufficient within-team agreement and between-team differences. Equity gaps are population-level distributional outcomes.

### 3.2 Change communication: establishing an intelligible, credible, and contestable direction for transformation

#### 3.2.1 From information transmission to an action narrative open to scrutiny

Available research indicates that change communication encompasses more than information transmission; it connects public health problems, technological choices, role changes, risk boundaries, and feedback channels into an interpretable narrative for action (17–20). Effective messages should explain the gap in the current state, the proposed approach, implementation capability, enabling conditions, and value for affected groups, while allowing different stakeholders to question the proposed direction. Ambiguous, contradictory, or overly optimistic messages may amplify rumors and fragment interpretation. Communication quality should therefore be evaluated in terms of goal comprehension, information credibility, space for contestation, and responsiveness to feedback rather than the number of meetings or emails.

In digital public health, a transformation narrative should connect technological capability to a clearly defined population health problem. For example, a real-time surveillance platform should not be justified solely by “moving data to cloud infrastructure” or “increasing automation.” It should explain how information delays affect outbreak detection, resource allocation, or coverage of vulnerable populations, and should clarify which judgments remain uncertain, which data do not represent the overall population, and which decisions require professional and community deliberation.

#### 3.2.2 Organizational readiness for change, voice, and uncertainty management

Responses to change are jointly shaped by participation, fairness, internal organizational trust—including trust in management and in the change process—anticipated benefits, effects on work, and context. Resistance may reveal design deficiencies or implementation injustice rather than merely conservative attitudes (21). Readiness comprises cognitive, affective, and behavioral-intention dimensions and may differ across individuals, work units, and organizations (22). Organizational readiness for change more closely reflects members’ shared judgment of collective commitment and collective implementation efficacy than a stable preimplementation attribute (23). Incorporating dissent and counterexamples into initiative, understanding, enactment, and reflective dialog can help recalibrate commitment and action (24).

Frontline personnel are often the first to encounter missing data, workarounds, algorithmic false positives, and excluded populations. Anonymous issue repositories, cross-level meetings, data-quality reviews, community feedback panels, and explicit escalation pathways can convert dispersed anomalies into organizational cues. Whether communication fosters organizational readiness for change depends on whether reported concerns receive a response, whether feedback changes design and resource allocation, and whether those who speak up are protected.

#### 3.2.3 Symmetrical communication, multilevel leadership, and boundaries of applicability

Complex transformations require alignment of direction and an appropriate distribution of interpretive authority. One-way transmission is insufficient for addressing local knowledge and ambiguity; two-way, open, and responsive communication is more conducive to a shared direction that can be questioned (25, 26). Senior leaders are responsible for stabilizing public value and non-negotiable governance principles. Middle managers translate strategic language into roles and workflows. Technical leads explain limitations of data and models. Frontline personnel and community partners need channels through which they can redefine the problem.

Communication intensity is not equivalent to communication quality. Highly consistent messaging can suppress dissent, whereas conflicting signals from leaders at different levels can weaken credibility. This mechanism is most applicable to complex transformations requiring cross-level interpretation and continuous adjustment; organizational readiness for change or adoption quality cannot be inferred from the frequency of communications. Existing evidence primarily supports associations of communication processes with readiness, voice, and adoption. Longitudinal evidence directly linking these processes to public health system or population outcomes remains limited.

### 3.3 Collective Sensemaking: translating diverse interpretations into shared problem representations

#### 3.3.1 Cue extraction, identity negotiation, and shared problem representation

Collective sensemaking enables actors to develop provisional problem representations that guide action when information is incomplete, events are unexpected, or identities are challenged (27–29). Formal meetings, informal interactions, data visualizations, prototypes, and other material objects can transform dispersed cues into shared interpretations. However, which interpretations become dominant depends on who has the authority to assign meaning, which forms of knowledge are treated as valid, and whether open contestation is permitted (30).

The central ambiguity in digital public health often concerns not whether a technology functions, but what the data and technology signify. A decline in case counts may indicate reduced transmission, but it may also result from insufficient testing, interface failure, or declining coverage of vulnerable populations. High average performance may likewise conceal systematic error in critical subgroups. Epidemiologists, data engineers, managers, legal professionals, and community representatives must jointly examine alternative explanations before technical metrics can become actionable public health cues.

#### 3.3.2 Translation, narrative competition, and prospective Sensemaking

Middle managers and project leads occupy the interface between strategy and operations and often reconstruct the meaning of change through lateral interactions, everyday practices, and testing of rumors (31, 32). Competing narratives of progress, regression, and stability influence whether organizational members support, reshape, or resist change (33). Material practices such as sketches, prototypes, storyboards, and scenario cards can make implicit expectations visible and support shared imagination of possible futures (34).

Before formal procurement and deployment, prototypes, data journey maps, structured scenario analyses, and worst-case analyses can be used to make professional assumptions, target populations, risk boundaries, evidence-updating rules, and stopping conditions explicit. Sensemaking does not seek complete consensus; it seeks a minimally shared frame sufficient for coordinated action. Dominant narratives may be controlled by high-status groups and exclude community knowledge or minority interpretations. Evaluation should therefore consider the visibility of alternative explanations, documentation of dissent, and capacity for revision. Existing evidence is drawn largely from organizational and team research; direct support in interorganizational public health settings and for long-term outcomes requires further testing.

### 3.4 Cross-functional collaboration: embedding shared understanding in joint action

#### 3.4.1 Knowledge boundaries, professional language, and boundary objects

Different functions develop their own interpretive frames and “domains of expertise,” and knowledge does not flow naturally across departments (35). Collaboration requires participants to preserve their professional perspectives while also understanding those of others (36), supported by boundary objects that retain recognizable common features across professional and organizational contexts (37). Syntactic, semantic, and pragmatic boundaries require, respectively, a shared vocabulary, translation of meaning, and negotiation of interests. When innovation changes existing distributions of cost and power, information sharing alone is insufficient for knowledge transformation (38, 39).

In digital public health, data dictionaries, ontologies, case definitions, service blueprints, algorithm cards, privacy impact assessments, dashboard prototypes, and responsibility matrices can serve as boundary objects. Their value depends on whether they encourage participants to disclose assumptions, identify incompatible definitions, and document trade-offs, rather than on the mere existence of the documents.

#### 3.4.2 Expertise coordination, psychological safety, and conflict repair

Cross-functional performance depends on identifying who possesses which knowledge, mobilizing relevant expertise when needed, and applying it to the task (40). Shared goals, knowledge scaffolds, and dynamic task reconfiguration can temporarily bridge knowledge differences (41), whereas clarity of responsibility, predictability, and shared understanding provide integrative conditions for coordination (42). Leader inclusiveness, reporting protection, and response mechanisms provide team-level proximal support for raising objections, reporting anomalies, and receiving feedback. Team psychological safety, as a proximal moderating condition, may influence whether these supports are translated into reports of data defects, challenges to algorithms, acknowledgment of uncertainty, and help-seeking (43–45).

Collaboration should not seek to eliminate conflict. Instead, task conflict, value conflict, and relationship conflict should be distinguished, and mechanisms should be established for issue escalation, evidence review, independent ethics review, incident reporting, and after-action review. Team psychological safety is neither an outcome of communication quality nor a mediator of organizational capability. Its theoretical position is limited to moderating the relationship of leader inclusiveness, reporting protection, and response mechanisms with risk-detection behaviors.

#### 3.4.3 Relational coordination, shared accountability, and interorganizational governance

Interprofessional collaboration depends jointly on shared goals, shared knowledge, mutual respect, and formal governance arrangements (46, 47). The association between information sharing and team performance depends more on whether unique information is openly discussed and integrated than on the total amount of information shared (48). At the interorganizational level, initial conditions, institutional design, facilitative leadership, shared motivation, and capacity for joint action shape collaborative governance (49, 50). Network size, the density of interorganizational trust, goal consensus, and capability requirements determine whether participant-governed networks, lead-organization governance, or network administrative organizations are appropriate (51, 52).

Collaboration and information sharing are generally associated with knowledge integration, task performance, and shared accountability, but they also increase coordination costs and may produce diffuse responsibility, closed coalitions, or concentrated power. Regional or national platforms should specify responsibility for data control, analysis, model approval, public communication, and emergency decision-making. Representativeness, community decision rights, compensation for participation, vendor accountability and portability obligations, independent auditing, and exit control should be included in evaluations of collaboration. The coordinating function of network management cannot substitute for public accountability. Available evidence primarily supports associations between collaboration and coordination, knowledge integration, and accountability processes; direct evidence of an independent causal effect on population outcomes remains lacking.

### 3.5 Coupling of the three mechanisms, framework comparison, and cross-level transmission

#### 3.5.1 Recursive rather than linear organizational mechanisms

Communication, sensemaking, and collaboration are not three sequentially completed steps. Research on crisis management and risk communication shows that cognition, communication, coordination, control, and feedback are interdependent, requiring organizations to update shared situational awareness and risk interpretations continuously under uncertainty (53, 54). Communication provides an initial direction and space for contestation; sensemaking translates professional interpretations into shared problem representations; and collaboration reveals new constraints through pilots and workflows. These constraints feed back into communication, revise narratives, and update shared understanding. The quality of this cycle depends on whether negative information, workarounds, and equity harms enter organizational learning.

#### 3.5.2 Relationship to established implementation and sociotechnical frameworks

Established frameworks explain digital technology implementation from different perspectives. The Consolidated Framework for Implementation Research (CFIR) organizes multilevel determinants. The Nonadoption, Abandonment, Scale-up, Spread, and Sustainability (NASSS) framework emphasizes complex technologies and their long-term evolution. The integrated Promoting Action on Research Implementation in Health Services (i-PARIHS) framework emphasizes alignment among the innovation, recipients, context, and facilitation. The Exploration, Preparation, Implementation, Sustainment (EPIS) framework highlights the implementation life cycle. Sociotechnical models emphasize coupling among technology, people, processes, and institutions (13, 15, 55–59). Building on these approaches, the present framework adds an interactive-mechanism layer that connects change communication, collective sensemaking, and cross-functional collaboration to organizational capabilities, implementation processes, and multilevel outcomes (Table 2).

**Table 2:** Comparison of the present framework with established implementation and sociotechnical frameworks.

Framework	Primary analytical focus	Core constructs or stages	Relationship to power, feedback, and equity	Relationship to the present framework
Sociotechnical model	Coupling of technology, people, processes, organizations, and external institutions	Hardware and software; content; interfaces; people; processes; policies; regulations; monitoring feedback	Recognizes system coupling and feedback; power and community decision rights require separate elaboration	The present framework adopts the system-coupling perspective and adds three interactive mechanisms and stratified outcomes
Consolidated Framework for Implementation Research (CFIR)	Identify multilevel determinants of implementation	Intervention; outer setting; inner setting; individuals; implementation process	Can incorporate equity and local conditions, but does not center the three interactive mechanisms	The present framework connects its contextual constructs to mechanisms, capabilities, and multilevel outcomes
Nonadoption, Abandonment, Scale-up, Spread, and Sustainability (NASSS) framework	Explain nonadoption, abandonment, scale-up, spread, and sustainability of complex technologies	Problem; technology; value proposition; adopter system; organization; institutional environment; mutual adaptation	Emphasizes complexity and temporal evolution; representativeness and vendor power can be specified further	The present framework adds intraorganizational negotiation of meaning, cross-functional accountability, and equity governance
integrated Promoting Action on Research Implementation in Health Services (i-PARIHS) framework	Explain how innovations are adopted through facilitation in context	Innovation; recipients; context; facilitation	Facilitators continually adapt to context; cross-jurisdictional vendor governance is not central	The present framework specifies communication, sensemaking, and collaboration as observable processes
Exploration, Preparation, Implementation, Sustainment (EPIS) framework	Explain the implementation life cycle in public services	Exploration; preparation; implementation; sustainment; outer and inner contexts	Stages and cross-level boundaries are explicit; power and equity are not the central axis	The present framework retains the life-cycle perspective and adds recursive feedback and organizational-capability mediators
Present framework	Explain how shared understanding is translated into joint action and public health value	Contextual conditions → three mechanisms → mediating capabilities → implementation outcomes → organizational/system outcomes → population-level service and equity outcomes → population health outcomes	Distinguishes structural conditions such as pre-existing power distributions from cross-cutting governance practices such as authorization for participation, auditing, and appeals	An interactive-mechanism layer that complements established frameworks

The comparison delineates the analytical focus and complementary relationship of each framework. CFIR, Consolidated Framework for Implementation Research; NASSS, Nonadoption, Abandonment, Scale-up, Spread, and Sustainability; i-PARIHS, integrated Promoting Action on Research Implementation in Health Services; EPIS, Exploration, Preparation, Implementation, Sustainment.

Recent evidence indicates persistent limitations in the usability and testability of implementation frameworks and in their application to digital decision support, equity, and trial implementation (60–63). Research on adaptation, scale-up, sustainability, the scope of implementation research, and resource-constrained settings further emphasizes long-term embedding and contextual fit (64–66). Studies of digital implementation costs, combinations of multiple frameworks, hybrid trials, development of implementation strategies, and framework selection call for explicit justification of both resource costs and theoretical combinations (67–71). Collectively, this literature indicates that an interactive-mechanism layer should represent contextual conditions, organizational processes, capability formation, and feedback simultaneously so that it can be used to complement established frameworks.

#### 3.5.3 Seven-layer integrated mechanism framework

Structural contextual conditions define the operating boundaries of the three mechanisms. Leader inclusiveness, reporting protection, and response mechanisms provide team-level proximal support for risk-detection behaviors; team psychological safety moderates the relationship between these support conditions and anomaly reporting, voice, help-seeking, and assumption challenging. Risk-detection behaviors feed problem cues back into change communication, collective sensemaking, and cross-functional collaboration. Through distinct potential mediating capabilities, the three mechanisms are linked to implementation outcomes and organizational or system outcomes. Organizational or system changes may be associated with population-level service and equity outcomes through coverage, service accessibility, subgroup performance, resource allocation, and burden distribution. When these changes have sustained effects on risk exposure, service effectiveness, or disease processes, they may in turn be associated with avoidable morbidity, mortality, functional loss, or health gains. Outcome monitoring and community feedback also return to the organizational mechanisms and may trigger adjustments in collaboration and governance. Authorization for participation, community decision rights, maintenance of representativeness, resource compensation, vendor accountability, independent auditing, appeals, portability, and exit management constitute governance practices that cut across all levels and enable auditing of decision-making authority, vendor control, and burden distribution (Figure 1).

**Figure 1:**
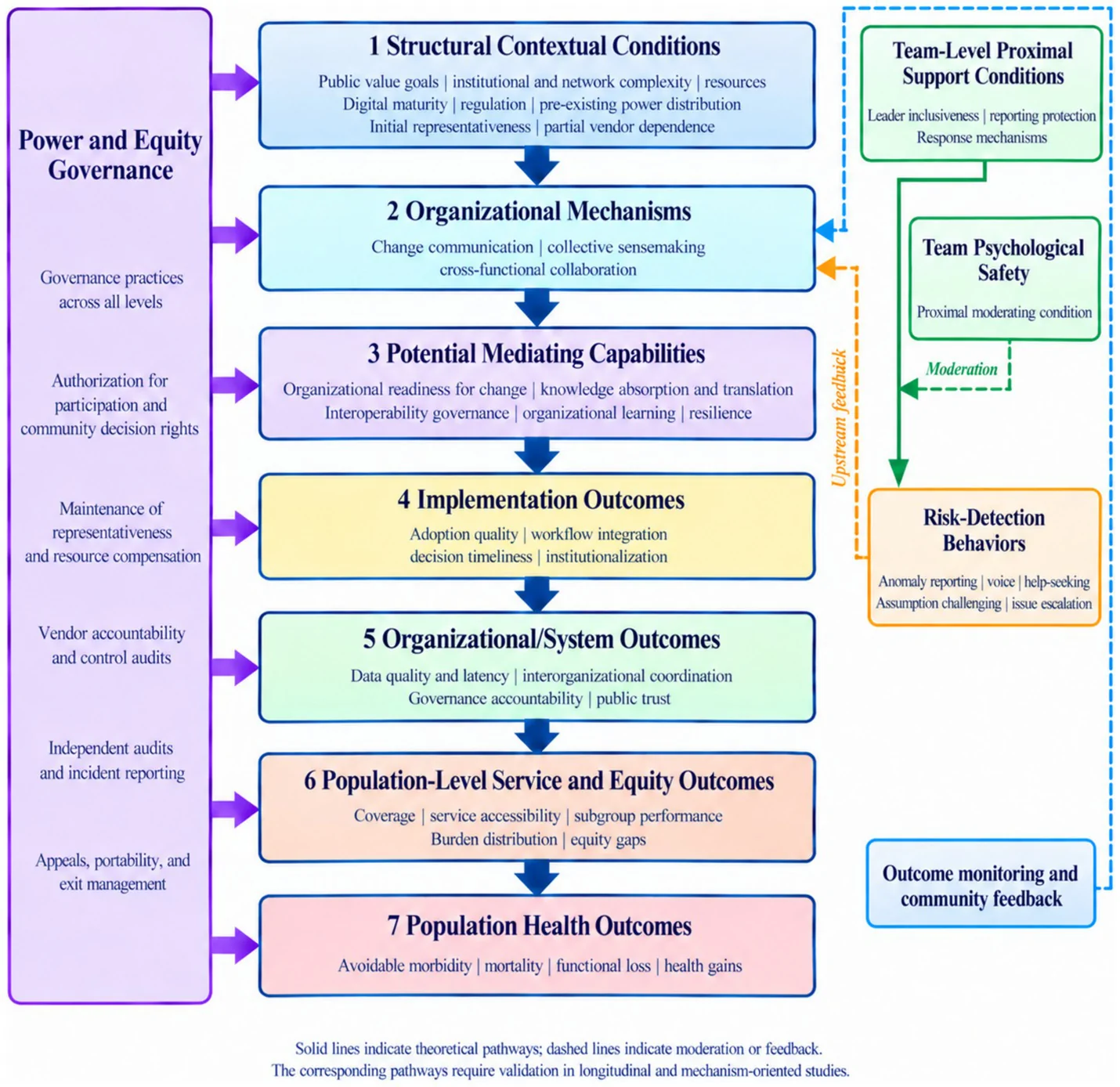
Seven-Layer integrated mechanism framework for digital public health transformation.

The seven layers are structural contextual conditions, organizational mechanisms, potential mediating capabilities, implementation outcomes, organizational or system outcomes, population-level service and equity outcomes, and population health outcomes. Leader inclusiveness, reporting protection, and response mechanisms constitute team-level proximal support conditions with a direct theoretical pathway to risk-detection behaviors. Team psychological safety moderates the relationship between these support conditions and risk-detection behaviors. Risk-detection behaviors feed problem cues back into the organizational mechanisms. Authorization for participation, community decision rights, maintenance of representativeness, resource compensation, vendor accountability, independent auditing, appeals, portability, and exit management form a cross-cutting governance axis. Solid lines indicate theoretical pathways, whereas dashed lines indicate moderation or feedback. The corresponding pathways require validation in longitudinal and mechanism-oriented studies.

#### 3.5.4 Cross-level transmission mechanisms

Cross-level transmission in digital public health transformation can be divided into five links. First, leader inclusiveness, reporting protection, and response mechanisms provide proximal support, and team psychological safety moderates the relationship of this support with anomaly reporting, voice, help-seeking, and assumption challenging. Individual perceptions of communication consistency and information credibility may also influence collective commitment. Second, through interaction, shared problem representation, and role coordination, teams aggregate individual responses and problem cues into team learning and organizational readiness for change. Individual-level measures should be aggregated to team- or organizational-level constructs only when they demonstrate sufficient within-team agreement and discernible between-team differences. Otherwise, analysis should remain at the individual level and multilevel models should be used to estimate cross-level mediation or moderation. Third, organizations translate team understanding into potential mediating capabilities—including knowledge absorption and translation, interoperability governance, organizational learning, and resilience—through standards, workflows, resource allocation, and accountability arrangements. Fourth, interorganizational networks use shared data definitions, interfaces, joint decision-making, and vendor governance to produce implementation outcomes such as adoption quality, workflow integration, timeliness of decision-making, and institutionalization, as well as organizational or system outcomes such as data quality, coordination timeliness, governance accountability, and public trust. Fifth, through resource allocation, service pathways, and risk exposure, these system processes affect coverage, service accessibility, subgroup performance, burden distribution, and equity gaps across populations, and may become associated with morbidity, mortality, functional loss, or health gains when they produce sustained changes in disease risk or service effectiveness. Anomalies, delays, burden distributions, and community appeals trigger feedback to the organizational mechanisms, including renewed change communication and collective sensemaking, and may prompt adjustments in collaboration and governance. This chain is intended to generate cross-level hypotheses; causal interpretations require longitudinal or quasi-experimental validation.

#### 3.5.5 Mechanism emphasis across the life cycle

Initiation and diagnosis, interpretation and co-production, piloting and integration, scale-up and interoperability, and institutionalization and learning constitute five interconnected organizational tasks. Each task involves communication, sensemaking, and collaboration; the relative emphasis of each mechanism is derived from theoretical synthesis. Pilot failure, external shocks, data-quality problems, vendor incidents, or equity risks may trigger return pathways and governance adjustments (Figure 2). The organizational requirements, risks, milestones, and mechanism emphases for each task are summarized accordingly (Tables 3 and 4).

**Figure 2:**
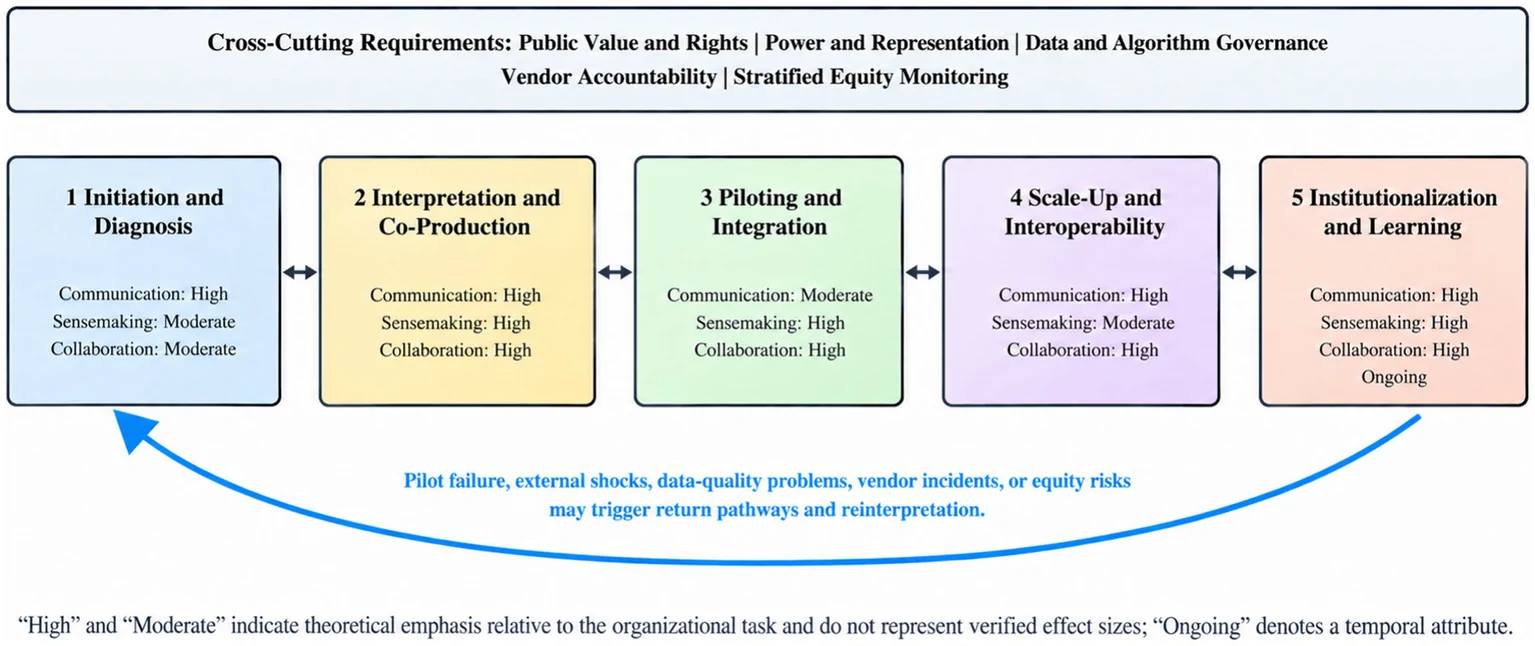
Recursive life cycle of digital public health transformation.

**Table 3:** Organizational tasks, risks, and milestones across the digital public health transformation life cycle.

Life-cycle task	Main organizational task	Communication focus	Sensemaking and collaboration arrangements	Principal risks and milestones
Initiation and diagnosis	Define the population health problem, beneficiary populations, value orientation, and constraints	Explain why transformation is needed, non-negotiable principles, and choices that remain open	Cross-professional diagnosis; map stakeholders and the data ecosystem; establish channels for voice	Risk: technology-first framing and problem displacement; milestone: shared problem statement and governance authorization
Interpretation and co-production	Translate professional and community knowledge into design hypotheses	Make uncertainty, expected effects, and trade-offs explicit	Prototypes, data journey maps, and structured scenario analyses; jointly define indicators and stopping rules	Risk: tokenistic participation and power asymmetry; milestone: traceable requirements and risk register
Piloting and integration	Embed the intervention in workflows, roles, data standards, and decisions	Continuously explain role changes, uses of feedback, and reasons for adjustment	Cross-functional team; boundary objects; rapid review and workflow redesign	Risk: workarounds and hidden errors; milestone: usability, data quality, and task completion
Scale-up and interoperability	Replicate across departments, jurisdictions, and vendors while maintaining comparability	Explain minimum common standards and the space for local adaptation	Network governance; coordinate standards, interfaces, responsibilities, and resources; cross-site learning	Risk: fragmentation or overstandardization; milestone: semantic comparability, clear accountability, and auditable adaptation
Institutionalization and learning	Incorporate monitoring, updating, auditing, and capability development into routine governance	Report value, limitations, incidents, and equity impacts	Joint performance management; update models and rules; community oversight; after-action review	Risk: treating deployment as the endpoint and technological lock-in; milestone: sustained use, improved outcomes, equity impacts, and continuity of core functions

**Table 4:** Matrix of the relative emphasis of the three mechanisms across five organizational tasks.

Life-cycle task	Change communication	Collective sensemaking	Cross-functional collaboration	Theoretical rationale for emphasis and feedback
Initiation and diagnosis	High: explain public value, problem boundaries, and non-negotiable principles	Moderate: form an initial problem representation while preserving alternative explanations	Moderate: establish stakeholder and data-ecosystem maps	Communication provides direction and legitimacy; sensemaking and collaboration provide diagnostic correction; power auditing begins here
Interpretation and co-production	High: make uncertainty, impacts, and trade-offs explicit	High: jointly extract cues, develop prototypes, and conduct structured scenario analyses	High: translate community, professional, and technical knowledge into design hypotheses	The three mechanisms jointly form a minimally shared frame; records of dissent trigger reinterpretation
Piloting and integration	Moderate: explain role changes, uses of feedback, and reasons for adjustment	High: test workflows, indicators, and risk boundaries	High: embed roles, interfaces, data standards, and joint decisions	Collaboration translates shared understanding into workflows; pilot anomalies feed back to communication and sensemaking
Scale-up and interoperability	High: explain minimum common standards and space for local adaptation	Moderate: compare jurisdictional definitions, rules, and equity impacts	High: coordinate interfaces, responsibilities, resources, and vendor relationships	Scale-up depends on common standards and controlled adaptation; cross-jurisdictional differences trigger shared interpretation
Institutionalization and learning	High: continuously report value, limitations, incidents, and equity impacts	High: continuously update problem representations, rules, and risk boundaries	High: continuously incorporate monitoring, auditing, training, and capability updates into routine governance	All three mechanisms sustain learning through feedback; major changes can trigger a return in the life cycle

“High” and “Moderate” indicate theoretical emphasis relative to each organizational task and do not represent effect sizes; “Ongoing” denotes a temporal attribute.

The five categories of organizational tasks are connected by bidirectional relationships and return pathways. “High” and “Moderate” indicate theoretical emphasis relative to each organizational task and do not represent verified effect sizes, whereas “Ongoing” denotes a temporal attribute. Pilot failure, external shocks, data-quality problems, vendor incidents, or equity risks may trigger renewed communication, sensemaking, and adjustments to collaboration.

### 3.6 Organizational capabilities as mediators between the three mechanisms and multilevel outcomes

#### 3.6.1 Dynamic capabilities, absorptive capacity, and organizational learning

Whether the three mechanisms extend beyond a one-off project depends on whether they are translated into repeatedly deployable organizational capabilities. Digital transformation requires continuous sensing of opportunities, resource allocation, reconfiguration of routines, and development of external collaboration (72, 73). Absorptive capacity depends on prior knowledge and boundary-spanning communication, enabling organizations to recognize, assimilate, and apply external knowledge (74). Organizational learning must also maintain tension between exploring new technologies and stabilizing existing services (75). Human agency and technological affordances form new routines through repeated interaction; digital capabilities should therefore accumulate through real work settings, reflection, and workflow redesign rather than be measured solely by training hours (76).

The five mediating capabilities lie between organizational mechanisms and outcomes. Organizational readiness for change, knowledge absorption and translation, interoperability governance, organizational learning, and resilience describe whether an organization can sustain action, learn across boundaries, coordinate data and responsibility, revise institutional arrangements, and maintain core functions. They are not population health outcomes.

#### 3.6.2 Interoperability and learning public health systems

Interoperability is not limited to interface compatibility; it also requires organizations to establish shared agreements on the meaning, use, authorization, and responsibility associated with data. Syntactic, semantic, process, and organizational interoperability must be integrated with governance, incentives, and work practices (77). Open standards can support interoperable connections, but identity, authorization, data-quality, and accountability rules determine whether data exchange is trustworthy (78). Learning systems require data to arise from practice, knowledge to be generated rapidly, and findings to feed back into action (79). Recent research on public health data systems and digital maturity further indicates that governance, workforce, infrastructure, interoperability, and predictive-analytics capabilities often develop unevenly, and that strategic visions may remain at the level of principle because implementation, maintenance, and oversight are insufficient (80–82).

Platform maturity should not be evaluated solely by the number of connected organizations. Implementation outcomes include adoption quality, workflow integration, timeliness of decision-making, and institutionalization. Organizational or system outcomes include data completeness, latency, interorganizational coordination, governance accountability, and public trust. Population-level service and equity outcomes include coverage, service accessibility, burden distribution, and equity gaps. Population health outcomes include avoidable morbidity, mortality, functional loss, and health gains. Organizational capabilities may connect these levels, but current evidence is insufficient to establish an independent causal effect for every link.

### 3.7 Operation of the mechanisms in major application contexts

#### 3.7.1 Digital surveillance, data infrastructures, and cross-jurisdictional platforms

Digital surveillance and contact tracing require both timeliness and legitimacy. Ethical design should specify necessity, proportionality, effectiveness, time limits, transparency, and the right to opt out (83), whereas delays in testing and tracing reduce the effectiveness of control measures (84). This evidence suggests that interorganizational information flows, role clarity, and timely coordination are components of technological usability. However, modeling results or findings from a single project cannot directly establish long-term population health effects.

Public-health-specific evidence shows that digital innovation has encompassed surveillance, risk communication, and contact tracing, but implementation evaluation, infrastructure, legal frameworks, and adoption quality vary substantially (85, 86). Modernization of public health data systems and surveillance networks based on electronic health records may improve updating and coverage, but remain constrained by missing data, inconsistent definitions, authorization boundaries, and dispersed responsibility (80, 87). Laboratory and mobile data-collection platforms further indicate that data standards, laboratory processes, local work practices, and joint interpretation must be coordinated simultaneously (88). Statewide digital-maturity assessments and reviews of system integration in resource-constrained countries further reveal that organizational readiness for change may exceed infrastructure and interoperability capabilities, and that strategic goals may be difficult to realize because of parallel systems, weak implementation, and vendor dependence (81, 82).

Recent research places system maturity, data infrastructure, workforce capability, digital equity, and interdisciplinary education within a common evaluative perspective. Studies of educational, healthcare, and digital public health capabilities treat AI and algorithmic literacy as important capacities for participation in governance, although direct evidence from public health practice remains limited (89–96). This evidence supports separate evaluation of governance, interoperability, workforce capability, community participation, and distributional outcomes, rather than replacing analysis of mechanisms and contexts with a single composite maturity score.

Studies of surveillance networks and laboratory platforms primarily describe data flows, joint interpretation, and interorganizational coordination (85–88), whereas research on maturity, capability, and education primarily delineates system contexts and capability gaps (81, 82, 89–96). In these settings, communication should explain the purpose of data collection, retention periods, individual and collective benefits, and opt-out rights. Sensemaking should determine whether changes in signals reflect epidemiological change or the data-generating process. Collaboration should connect laboratories, surveillance personnel, local government, vendors, legal professionals, and community organizations. Existing evidence provides stronger support for organizational and system processes than for long-term causal effects on population health.

#### 3.7.2 Artificial intelligence and algorithmic governance

AI governance should cover problem definition, data selection, model development, validation, deployment integration, continuous monitoring, updating, and retirement. Research on ethics and responsible machine learning emphasizes autonomy, safety, transparency, accountability, inclusion, and life-cycle hazard identification (97–99). Regulatory considerations from the World Health Organization further extend governance to risk classification, documentation, and post-deployment obligations (100). Research on large language models identifies risks related to factual reliability, scope of applicability, racialized bias, and health equity (101–106). For regulated AI-enabled medical-device software functions, the predetermined change control plan of the United States Food and Drug Administration establishes requirements for model updating and change management (107). International Organization for Standardization/International Electrotechnical Commission (ISO/IEC) 42,001 and ISO/IEC 23894 provide references for organizational management and risk management (108, 109). Research on foundation models, implementation pathways, and public health governance further emphasizes transfer across tasks, institutional responsibility, and organizational integration (110–112). Health-system governance, proactive monitoring, bias mitigation, and governance documents from the World Health Organization, the United States National Institute of Standards and Technology, and the European Union jointly specify deployment review, human oversight, and continuous risk management (113–119). Research agendas, regulatory oversight, impact assessment, fairness methods, institutional guidance, data governance, and implementation-review frameworks further elaborate the accountability chain (120–128).

Public health policy research emphasizes that AI governance should be connected to institutional missions, public value, and population risk (129). Post-deployment studies in healthcare institutions show that erroneous outputs and their clinical consequences require ongoing monitoring (130). Consensus guidance on trustworthy AI, reporting standards, clinical decision support, deployment monitoring, implementation at scale, and allocation of responsibility further recommends transparent reporting, external validation, stratified performance monitoring, change control, human review, incident reporting, and decommissioning mechanisms (131–136). This evidence is used primarily to delineate governance and monitoring requirements.

The three mechanisms perform distinct functions across the AI life cycle. Change communication explains intended uses, prohibited uses, data sources, limitations, human review, and channels for appeal. Collective sensemaking combines model outputs with epidemiological evidence, social context, and community experience to identify situations in which “average performance is good but specific subgroups are harmed” or “predictions are accurate but not actionable.” Cross-functional collaboration brings epidemiology, statistics, engineering, ethics, law, communities, and vendors into shared governance and specifies responsibilities for deployment, monitoring, updating, and retirement (120, 123, 125, 128, 129, 131, 134–136).

#### 3.7.3 Digital public participation, power, and health equity

Digital transformation may increase coverage, but it may also translate social inequalities into inequalities in data and services. Technological access, capacity for use, data representativeness, social conditions, and resource redistribution shape equity outcomes (137–142). Insufficient digital readiness may further limit access to digital tools and equitable benefits through constrained implementation capability and sustained support (143). Co-production can connect local knowledge with scientific practice, but whether it moves beyond tokenistic participation depends on whether power, resources, and accountability are substantively transferred (144, 145). A recent synthesis of AI and digital health equity likewise indicates that equitable benefits depend on enabling access, subgroup validation, accountable governance, and implementation oversight rather than on the technology itself (146).

Community co-production should encompass problem definition, data selection, interpretation of results, risk trade-offs, resource allocation, and governance oversight. Evaluation indicators include representativeness, actual decision-making authority, resources and compensation for participation, closed feedback loops, vendor influence, stratified performance, and burden distribution. Procurement and partnership agreements should also specify data ownership, portability of models and interfaces, vendor obligations for updates and exit, independent auditing, incident notification, channels for appeal, and disclosure of conflicts of interest. Equity gaps are population-level service and equity outcomes, whereas equity governance is an audit requirement that cuts across all levels.

### 3.8 Research agenda and implications for practice

#### 3.8.1 From training programs to organizational digital readiness

One-off training is insufficient to meet digital capability-development needs. Public health competency frameworks treat data science, informatics, ethics, communication, implementation, interdisciplinary collaboration, and leadership as interrelated capabilities (9–11). Training, leadership support, resources, peer influence, work relevance, and sustained technical support jointly shape readiness; isolated courses cannot overcome constraints related to time, infrastructure, and culture (143). Public health agencies can develop data and ethics literacy at the role level, interprofessional problem-solving capacity and team conditions that support psychological safety at the team level, and governance, procurement, standards, learning, and workforce planning at the organizational level. Digital-maturity assessments should be linked to actual tasks and capability gaps rather than used only to generate aggregate rankings (81, 82).

#### 3.8.2 Reframing evaluation through equity and co-production

Evaluation should track technological access, social conditions, institutional design, and the distribution of power simultaneously (142). The quality of co-production depends on whether communities have actual decision-making authority over problem definition, data selection, interpretation, risk trade-offs, and oversight, and whether participation is adequately resourced and compensated (144, 145). For AI and other rapidly evolving technologies, subgroup performance, digital exclusion, appeals, vendor control, and burden distribution should also be documented continuously (146).

#### 3.8.3 A testable multilevel research agenda

Future research should move from asking “which factors are associated with transformation?” to asking “which mechanisms produce which outcomes, through which levels, and under what conditions?” Longitudinal and multilevel studies should simultaneously measure individual perceptions of communication; team-level shared problem representations and psychological safety; organizational readiness for change and workflows; interorganizational interoperability governance; system-process outcomes; and population service, equity, and health outcomes. Policy changes, staggered implementation, natural experiments, and process tracing can be used to test aggregation, mediation, moderation, and feedback. Population outcomes should be connected to organizational processes through resource allocation, service pathways, coverage, and risk exposure. Priority research questions, propositions, cross-level pathways, designs, and outcomes are presented (Table 5).

**Table 5:** Multilevel research questions, testable propositions, and applicable designs.

Research question	Testable proposition	Cross-level transmission	Applicable design	Principal outcomes
How does communication generate readiness?	Specific, symmetrical communication that acknowledges uncertainty may increase readiness through credibility and collective efficacy; its effect is conditioned by power structures and resource conditions	Individual perceptions → voice/collective commitment → team aggregation → organizational readiness for change	Longitudinal surveys; staggered implementation; multilevel modeling	Goal comprehension; responsiveness to feedback; collective commitment; adoption quality
When does shared meaning facilitate implementation?	When tasks are highly interdependent and knowledge differences are substantial, shared problem representations may facilitate collaboration; the effect may weaken when high-status groups dominate	Team cue extraction → shared representation → standards/workflows → interorganizational coordination	Process tracing; semantic analysis of meetings and documents; network analysis	Alternative explanations; rework rate; decision consistency
How does psychological safety moderate risk detection?	Leader inclusiveness, reporting protection, and response mechanisms provide proximal support; team psychological safety may moderate the effects of this support on anomaly reporting and voice, whereas corrective responses determine whether the information is translated further into organizational learning	Proximal support conditions × team psychological safety → anomaly reporting/voice → corrective response → organizational learning	Longitudinal team studies; multilevel moderation analysis; natural experiments	Anomaly reporting; correction time; voice; help-seeking
How should governance modes be matched to network conditions?	When networks are large, trust is low, and capability requirements are high, a dedicated coordinating organization may improve coordination, but concentration of power must be prevented	Organizational roles → common standards/interfaces → network governance → system accountability	Comparative case studies; network analysis; configurational comparison	Timeliness of coordination; legitimacy; auditing; sustainability
How do mechanisms affect equity?	Community co-production and stratified monitoring may reduce disparities; insufficient representativeness or vendor control may produce the opposite result	Community decision rights → data selection/interpretation → resource allocation → stratified service outcomes	Participatory research; quasi-experiments; distributional-effect analysis	Coverage gaps; subgroup performance; burden distribution; health gains
Do organizational tasks alter mechanism emphasis?	Different organizational tasks may alter the relative emphasis of the three mechanisms, and feedback may trigger a return to earlier tasks	Task → mechanism configuration → milestone/feedback → reinterpretation	Intensive longitudinal designs; dynamic structural models	Task milestones; delays; adaptation; institutionalization
Can digital capabilities generate long-term public value?	Organizational learning and interoperability governance may connect short-term adoption to long-term system or population outcomes, but direct causal evidence is limited	Organizational capability → system process → service and equity outcomes → health outcomes/feedback	Long-term follow-up; realist evaluation; system dynamics	Data quality; continuity of core functions; coverage; equity gaps; sustainability

The propositions in this table are intended to generate testable hypotheses for subsequent research.

## 4 Discussion

Synthesis of the available evidence indicates that the three mechanisms are better understood as recursively interacting organizational processes than as a fixed sequence of “communication first, sensemaking second, and collaboration last.” Communication can establish direction and channels for voice, but ambiguous, conflicting, or overly optimistic messages can increase uncertainty. Sensemaking can create shared problem representations, but dominant narratives may exclude minority groups and community knowledge. Collaboration can improve knowledge integration and task coordination, but coordination costs and power asymmetries may weaken its benefits (17, 20, 21, 23, 27, 29, 30, 34, 43, 45, 48, 51). The three mechanisms are more likely to influence implementation and system processes through organizational capabilities when representativeness, resources, auditing, and corrective mechanisms are clearly established.

The framework is theoretically grounded in established implementation and sociotechnical frameworks. CFIR provides multilevel contexts and determinants; NASSS emphasizes complex technologies and sustainability; i-PARIHS foregrounds facilitation; and EPIS organizes the implementation life cycle (55–59). Recent studies further emphasize theoretical justification for framework selection, usability, testability, equity, implementation costs, adaptation to digital technologies, and combinations of multiple frameworks (60–71). Change communication, collective sensemaking, and cross-functional collaboration are defined as observable interactive processes and linked to organizational readiness for change, knowledge absorption and translation, interoperability governance, organizational learning, and resilience. The seven-layer pathway and life-cycle matrix still require longitudinal testing across contexts.

Governance should shift its focus from the number of deployed systems and one-time technical performance to auditable chains of responsibility. Research on public health data systems, digital maturity, digital equity, and the workforce shows that interoperability, governance, infrastructure, workforce capability, and conditions of access may develop unevenly (81, 89–96). AI governance in public health also requires institutional missions, public participation, harm mitigation, and post-deployment oversight to be embedded in routine arrangements (100–112). Synthesis of equity research further indicates that subgroup validation, conditions of access, and accountable governance influence whether technology narrows or widens disparities (146). Organizations should regularly report who participated in problem definition, which views changed the design, who controls models and interfaces, and how anomalous or inequitable outcomes are reported and corrected, and how systems are withdrawn when necessary.

There is no universally optimal balance between speed and deliberation, standardization and local adaptation, centralized governance and distributed ownership, or data openness and privacy and security. Crisis settings may require rapid authorization and minimum common standards, but prolonged centralization may weaken local adaptation, public trust, and community control. Broad deliberation can strengthen legitimacy but may also delay urgent action. These trade-offs can be managed by prespecifying authorization boundaries, review triggers, the time point for restoring routine governance, and minimum equity safeguards (49, 50, 53, 54, 83, 127, 128).

Vendor control is an important boundary condition. Proprietary interfaces, nonportable data, opaque updating practices, and barriers to exit may lock public institutions into a single technological pathway. Procurement contracts and partnership agreements should translate auditing, data portability, incident notification, control of updates, exit obligations, and community oversight into enforceable responsibilities (15, 111, 116–119).

This review has five limitations. First, the three mechanisms continue to overlap theoretically and in their measurement across the existing literature. Although the analytical positions of psychological safety, organizational readiness for change, internal organizational trust, interorganizational trust, and public trust are distinguished here, operationalization may still vary across levels between studies. Second, the evidence spans organizational science, implementation science, and digital public health, with heterogeneous study designs, contexts, and outcomes; a common effect estimate cannot be derived. Third, longitudinal causal evidence directly from public health surveillance, laboratory networks, and cross-jurisdictional platforms remains limited, and some interpretations are drawn from health services and general organizational research. Fourth, evidence from resource-constrained settings, community-led transformation, and the power of commercial vendors is insufficient. Fifth, generative AI, foundation models, and regulatory frameworks are evolving rapidly. Recent literature can inform governance principles but cannot substitute for long-term, cross-jurisdictional, and population-equity evaluations. At present, the framework should therefore be regarded as a testable explanatory structure and governance-diagnostic tool rather than a universally applicable fixed causal model.

## 5 Conclusion

Available evidence suggests that digital public health transformation may depend on whether organizations can establish a credible direction, shared problem representations, and coordinated action. Through organizational readiness for change, knowledge absorption and translation, interoperability governance, organizational learning, and resilience, the three mechanisms may be associated with implementation outcomes, organizational or system outcomes, and population-level service and equity outcomes. Their pathways to population health outcomes require testing in longitudinal and mechanism-oriented studies. Practice evaluation should simultaneously address community decision rights, representativeness, resource compensation, vendor accountability, interoperability, organizational learning, and equity impacts.

## References

[1] Iyamu I Xu AXT Gómez-Ramírez O Ablona A Chang HJ Mckee G . Defining digital public health and the role of digitization, digitalization, and digital transformation: scoping review. JMIR Public Health Surveill. (2021) 7:e30399. doi: 10.2196/30399, 34842555 PMC8665390

[2] World Health Organization. Global Strategy on Digital Health 2020–2025. Geneva: World Health Organization (2021).

[3] Kostkova P. Grand challenges in digital health. Front Public Health. (2015) 3:134. doi: 10.3389/fpubh.2015.00134, 26000272 PMC4419711

[4] Kickbusch I Piselli D Agrawal A Balicer R Banner O Adelhardt M . The lancet and financial times commission on governing health futures 2030: growing up in a digital world. Lancet. (2021) 398:1727–76. doi: 10.1016/S0140-6736(21)01824-9, 34706260

[5] Vial G. Understanding digital transformation: a review and a research agenda. J Strateg Inf Syst. (2019) 28:118–44. doi: 10.1016/j.jsis.2019.01.003

[6] Hinings B Gegenhuber T Greenwood R. Digital innovation and transformation: an institutional perspective. Inf Organ. (2018) 28:52–61. doi: 10.1016/j.infoandorg.2018.02.004

[7] Mergel I Edelmann N Haug N. Defining digital transformation: results from expert interviews. Gov Inf Q. (2019) 36:101385. doi: 10.1016/j.giq.2019.06.002

[8] Werner L Puta C Chilalika T Walker Hyde S Cooper H Goertz H . How digital transformation can accelerate data use in health systems. Front Public Health. (2023) 11:1106548. doi: 10.3389/fpubh.2023.1106548, 37006561 PMC10050688

[9] Ramachandran S Chang HJ Worthington C Kushniruk A Ibáñez-Carrasco F Davies H . Digital competencies and training approaches to enhance the capacity of practitioners to support the digital transformation of public health: rapid review of current recommendations. JMIR Public Health Surveill. (2024) 10:e52798. doi: 10.2196/52798, 39248660 PMC11403915

[10] Hrzic R Buttigieg S Wong BLH Silva AM Conyard KF Codd M . Reflecting digital transformations in public health curricula. Public Health Rev. (2025) 46:1608237. doi: 10.3389/phrs.2025.1608237, 40144365 PMC11936747

[11] Schmidt J Duplaga M Le Meur N Wong BLH Maaß L Malešević A . A competency framework for digital public health in Europe: an updated scoping review. npj Digit Public Health. (2026) 1:18. doi: 10.1038/s44482-026-00023-9

[12] Budd J Miller BS Manning EM Lampos V Zhuang M Edelstein M . Digital technologies in the public-health response to COVID-19. Nat Med. (2020) 26:1183–92. doi: 10.1038/s41591-020-1011-4 32770165

[13] Sittig DF Singh H. A new sociotechnical model for studying health information technology in complex adaptive healthcare systems. Qual Saf Health Care. (2010) 19:i68–74. doi: 10.1136/qshc.2010.042085, 20959322 PMC3120130

[14] Cresswell KM Sheikh A. Organizational issues in the implementation and adoption of health information technology innovations: an interpretative review. Int J Med Inform. (2013) 82:e73–86. doi: 10.1016/j.ijmedinf.2012.10.007, 23146626

[15] Greenhalgh T Wherton J Papoutsi C Lynch J Hughes G A’Court C . Beyond adoption: a new framework for theorizing and evaluating nonadoption, abandonment, and challenges to the scale-up, spread, and sustainability of health and care technologies. J Med Internet Res. (2017) 19:e367. doi: 10.2196/jmir.8775, 29092808 PMC5688245

[16] Orlikowski WJ. The duality of technology: rethinking the concept of technology in organizations. Organ Sci. (1992) 3:398–427. doi: 10.1287/orsc.3.3.398, 19642375

[17] Lewis LK. An organizational stakeholder model of change implementation communication. Commun Theory. (2007) 17:176–204. doi: 10.1111/j.1468-2885.2007.00291.x

[18] Armenakis AA Harris SG. Crafting a change message to create transformational readiness. J Organ Change Manag. (2002) 15:169–83. doi: 10.1108/09534810210423080

[19] Elving WJL. The role of communication in organisational change. Corp Commun. (2005) 10:129–38. doi: 10.1108/13563280510596943

[20] Bordia P Hobman E Jones E Gallois C Callan VJ. Uncertainty during organizational change: types, consequences, and management strategies. J Bus Psychol. (2004) 18:507–32. doi: 10.1023/B:JOBU.0000028449.99127.f7

[21] Oreg S Vakola M Armenakis A. Change recipients’ reactions to organizational change: a 60-year review of quantitative studies. J Appl Behav Sci. (2011) 47:461–524. doi: 10.1177/0021886310396550

[22] Rafferty AE Jimmieson NL Armenakis AA. Change readiness: a multilevel review. J Manage. (2013) 39:110–35. doi: 10.1177/0149206312457417, 42711695

[23] Weiner BJ. A theory of organizational readiness for change. Implement Sci. (2009) 4:67. doi: 10.1186/1748-5908-4-67, 19840381 PMC2770024

[24] Ford JD Ford LW. The role of conversations in producing intentional change in organizations. Acad Manag Rev. (1995) 20:541–70. doi: 10.5465/amr.1995.9508080330

[25] Johansson C Heide M. Speaking of change: three communication approaches in studies of organizational change. Corp Commun. (2008) 13:288–305. doi: 10.1108/13563280810893661

[26] Sun R Li JYQ Lee Y Tao W. The role of symmetrical internal communication in improving employee experiences and organizational identification during COVID-19 pandemic-induced organizational change. Int J Bus Commun. (2023) 60:1398–426. doi: 10.1177/23294884211050628

[27] Gioia DA Chittipeddi K. Sensemaking and sensegiving in strategic change initiation. Strateg Manag J. (1991) 12:433–48. doi: 10.1002/smj.4250120604

[28] Weick KE Sutcliffe KM Obstfeld D. Organizing and the process of sensemaking. Organ Sci. (2005) 16:409–21. doi: 10.1287/orsc.1050.0133

[29] Maitlis S Christianson M. Sensemaking in organizations: taking stock and moving forward. Acad Manag Ann. (2014) 8:57–125. doi: 10.5465/19416520.2014.873177

[30] Maitlis S Lawrence TB. Triggers and enablers of sensegiving in organizations. Acad Manag J. (2007) 50:57–84. doi: 10.5465/amj.2007.24160971

[31] Balogun J Johnson G. Organizational restructuring and middle manager sensemaking. Acad Manag J. (2004) 47:523–49. doi: 10.5465/20159600

[32] Rouleau L. Micro-practices of strategic sensemaking and sensegiving: how middle managers interpret and sell change every day. J Manag Stud. (2005) 42:1413–41. doi: 10.1111/j.1467-6486.2005.00549.x

[33] Sonenshein S. We’re changing-or are we? Untangling the role of progressive, regressive, and stability narratives during strategic change implementation. Acad Manag J. (2010) 53:477–512. doi: 10.5465/amj.2010.51467638

[34] Stigliani I Ravasi D. Organizing thoughts and connecting brains: material practices and the transition from individual to group-level prospective sensemaking. Acad Manag J. (2012) 55:1232–59. doi: 10.5465/amj.2010.0890

[35] Dougherty D. Interpretive barriers to successful product innovation in large firms. Organ Sci. (1992) 3:179–202. doi: 10.1287/orsc.3.2.179

[36] Boland RJ Jr Tenkasi RV. Perspective making and perspective taking in communities of knowing. Organ Sci. (1995) 6:350–72. doi: 10.1287/orsc.6.4.350, 19642375

[37] Star SL Griesemer JR. Institutional ecology, ‘translations’ and boundary objects: amateurs and professionals in Berkeley’s Museum of Vertebrate Zoology, 1907-39. Soc Stud Sci. (1907) 19:387–420. doi: 10.1177/030631289019003001

[38] Carlile PR. A pragmatic view of knowledge and boundaries: boundary objects in new product development. Organ Sci. (2002) 13:442–55. doi: 10.1287/orsc.13.4.442.2953, 19642375

[39] Carlile PR. Transferring, translating, and transforming: an integrative framework for managing knowledge across boundaries. Organ Sci. (2004) 15:555–68. doi: 10.1287/orsc.1040.0094, 19642375

[40] Faraj S Sproull L. Coordinating expertise in software development teams. Manag Sci. (2000) 46:1554–68. doi: 10.1287/mnsc.46.12.1554.12072, 19642375

[41] Majchrzak A More PHB Faraj S. Transcending knowledge differences in cross-functional teams. Organ Sci. (2012) 23:951–70. doi: 10.1287/orsc.1110.0677, 19642375

[42] Okhuysen GA Bechky BA. Coordination in organizations: an integrative perspective. Acad Manage Ann. (2009) 3:463–502. doi: 10.1080/19416520903047533

[43] Edmondson AC. Psychological safety and learning behavior in work teams. Adm Sci Q. (1999) 44:350–83. doi: 10.2307/2666999

[44] Nembhard IM Edmondson AC. Making it safe: the effects of leader inclusiveness and professional status on psychological safety and improvement efforts in health care teams. J Organ Behav. (2006) 27:941–66. doi: 10.1002/job.413

[45] Frazier ML Fainshmidt S Klinger RL Pezeshkan A Vracheva V. Psychological safety: a meta-analytic review and extension. Pers Psychol. (2017) 70:113–65. doi: 10.1111/peps.12183

[46] D’Amour D Goulet L Labadie JF Martín-Rodriguez LS Pineault R. A model and typology of collaboration between professionals in healthcare organizations. BMC Health Serv Res. (2008) 8:188. doi: 10.1186/1472-6963-8-188, 18803881 PMC2563002

[47] Gittell JH Godfrey M Thistlethwaite J. Interprofessional collaborative practice and relational coordination: improving healthcare through relationships. J Interprof Care. (2013) 27:210–3. doi: 10.3109/13561820.2012.730564, 23082769

[48] Mesmer-Magnus JR DeChurch LA. Information sharing and team performance: a meta-analysis. J Appl Psychol. (2009) 94:535–46. doi: 10.1037/a0013773, 19271807

[49] Ansell C Gash A. Collaborative governance in theory and practice. J Public Adm Res Theory. (2008) 18:543–71. doi: 10.1093/jopart/mum032

[50] Emerson K Nabatchi T Balogh S. An integrative framework for collaborative governance. J Public Adm Res Theory. (2012) 22:1–29. doi: 10.1093/jopart/mur011

[51] Bryson JM Crosby BC Stone MM. The design and implementation of cross-sector collaborations: propositions from the literature. Public Adm Rev. (2006) 66:44–55. doi: 10.1111/j.1540-6210.2006.00665.x, 40046247

[52] Provan KG Kenis P. Modes of network governance: structure, management, and effectiveness. J Public Adm Res Theory. (2008) 18:229–52. doi: 10.1093/jopart/mum015

[53] Comfort LK. Crisis management in hindsight: cognition, communication, coordination, and control. Public Adm Rev. (2007) 67:189–97. doi: 10.1111/j.1540-6210.2007.00827.x, 40046247

[54] Reynolds B Seeger MW. Crisis and emergency risk communication as an integrative model. J Health Commun. (2005) 10:43–55. doi: 10.1080/10810730590904571, 15764443

[55] Damschroder LJ Aron DC Keith RE Kirsh SR Alexander JA Lowery JC. Fostering implementation of health services research findings into practice: a consolidated framework for advancing implementation science. Implement Sci. (2009) 4:50. doi: 10.1186/1748-5908-4-50, 19664226 PMC2736161

[56] Damschroder LJ Reardon CM Widerquist MAO Lowery JC. The updated consolidated framework for implementation research based on user feedback. Implement Sci. (2022) 17:75. doi: 10.1186/s13012-022-01245-0, 36309746 PMC9617234

[57] Harvey G Kitson A. PARIHS revisited: from heuristic to integrated framework for the successful implementation of knowledge into practice. Implement Sci. (2016) 11:33. doi: 10.1186/s13012-016-0398-2, 27013464 PMC4807546

[58] Aarons GA Hurlburt M Horwitz SM. Advancing a conceptual model of evidence-based practice implementation in public service sectors. Admin Pol Ment Health. (2011) 38:4–23. doi: 10.1007/s10488-010-0327-7, 21197565 PMC3025110

[59] Hunter SC Kim B Kitson AL. Mobilising implementation of i-PARIHS (mi-PARIHS): development of a facilitation planning tool to accompany the integrated promoting action on research implementation in health services framework. Implement Sci Commun. (2023) 4:2. doi: 10.1186/s43058-022-00379-y, 36624543 PMC9830739

[60] Wang Y Wong ELY Nilsen P Chung VCH Tian Y Yeoh EK. A scoping review of implementation science theories, models, and frameworks—an appraisal of purpose, characteristics, usability, applicability, and testability. Implement Sci. (2023) 18:43. doi: 10.1186/s13012-023-01296-x, 37726779 PMC10507824

[61] Abell B Naicker S Rodwell D Donovan T Tariq A Baysari M . Identifying barriers and facilitators to successful implementation of computerized clinical decision support systems in hospitals: a NASSS framework-informed scoping review. Implement Sci. (2023) 18:32. doi: 10.1186/s13012-023-01287-y, 37495997 PMC10373265

[62] Gustafson P Abdul Aziz Y Lambert M Bartholomew K Rankin N Fusheini A . A scoping review of equity-focused implementation theories, models and frameworks in healthcare and their application in addressing ethnicity-related health inequities. Implement Sci. (2023) 18:51. doi: 10.1186/s13012-023-01304-0, 37845686 PMC10578009

[63] Williams A Lennox L Harris M Antonacci G. Supporting translation of research evidence into practice—the use of normalisation process theory to assess and inform implementation within randomised controlled trials: a systematic review. Implement Sci. (2023) 18:55. doi: 10.1186/s13012-023-01311-1, 37891671 PMC10612208

[64] Sun L Booth A Sworn K. Adaptability, scalability and sustainability (ASaS) of complex health interventions: a systematic review of theories, models and frameworks. Implement Sci. (2024) 19:52. doi: 10.1186/s13012-024-01375-7, 39020399 PMC11253497

[65] Wilson P Aarons GA Sales A Xu D Wensing M Hutchinson A . Revisiting the scope and expectations of implementation science and implementation science communications. Implement Sci. (2024) 19:69. doi: 10.1186/s13012-024-01399-z, 39367487 PMC11452926

[66] Tumuhimbise W Theuring S Kaggwa F Atukunda EC Rubaihayo J Atwine D . Enhancing the implementation and integration of mHealth interventions in resource-limited settings: a scoping review. Implement Sci. (2024) 19:72. doi: 10.1186/s13012-024-01400-9, 39402567 PMC11476919

[67] Donovan T Abell B McPhail SM Carter HE . Development of an instrument (cost-IS) to estimate costs of implementation strategies for digital health solutions: a modified e-Delphi study. Implement Sci. (2025) 20:13. doi: 10.1186/s13012-025-01423-w PMC11889902 40055802

[68] Hunter SC Morgillo S Kim B Bergström A Ehrenberg A Eldh AC . Combined use of the integrated-promoting action on research implementation in health services (i-PARIHS) framework with other implementation frameworks: a systematic review. Implement Sci Commun. (2025) 6:25. doi: 10.1186/s43058-025-00704-1, 40051001 PMC11887334

[69] Atzmon O Crowther ME Bei B O’Connor DA. The use of implementation science theoretical approaches in hybrid effectiveness-implementation type 1 randomised trials of healthcare interventions: a scoping review. Implement Sci. (2025) 20:23. doi: 10.1186/s13012-025-01435-6, 40380198 PMC12083051

[70] May CR Hillis A Albers B Desveaux L Gilbert A Girling M . Translational framework for implementation evaluation and research: implementation strategies derived from normalization process theory. Implement Sci. (2025) 20:34. doi: 10.1186/s13012-025-01444-5, 40717109 PMC12297445

[71] Fontaine G Mooney M Porat-Dahlerbruch J Cahir K Ellen M Spinewine A . Advancing the selection of implementation science theories, models, and frameworks: a scoping review and the development of the SELECT-IT meta-framework. Implement Sci. (2025) 20:24. doi: 10.1186/s13012-025-01436-5, 40437531 PMC12117738

[72] Warner KSR Wäger M. Building dynamic capabilities for digital transformation: an ongoing process of strategic renewal. Long Range Plan. (2019) 52:326–49. doi: 10.1016/j.lrp.2018.12.001

[73] Teece DJ Pisano G Shuen A. Dynamic capabilities and strategic management. Strateg Manag J. (1997) 18:509–33. doi: 10.1002/(SICI)1097-0266(199708)18:7<>3.0.CO;2-Z

[74] Cohen WM Levinthal DA. Absorptive capacity: a new perspective on learning and innovation. Adm Sci Q. (1990) 35:128–52. doi: 10.2307/2393553

[75] March JG. Exploration and exploitation in organizational learning. Organ Sci. (1991) 2:71–87. doi: 10.1287/orsc.2.1.71, 19642375

[76] Leonardi PM. When flexible routines meet flexible technologies: affordance, constraint, and the imbrication of human and material agencies. MIS Q. (2011) 35:147–67. doi: 10.2307/23043493

[77] Lehne M Sass J Essenwanger A Schepers J Thun S. Why digital medicine depends on interoperability. npj Digit Med. (2019) 2:79. doi: 10.1038/s41746-019-0158-1, 31453374 PMC6702215

[78] Mandel JC Kreda DA Mandl KD Kohane IS Ramoni RB. SMART on FHIR: a standards-based, interoperable apps platform for electronic health records. J Am Med Inform Assoc. (2016) 23:899–908. doi: 10.1093/jamia/ocv189, 26911829 PMC4997036

[79] Friedman CP Wong AK Blumenthal D. Achieving a nationwide learning health system. Sci Transl Med. (2010) 2:57cm29. doi: 10.1126/scitranslmed.3001456, 21068440

[80] Kadakia KT DeSalvo KB. Transforming public health data systems to advance the population’s health. Milbank Q. (2023) 101:674–99. doi: 10.1111/1468-0009.12618, 37096606 PMC10126962

[81] Snowdon A Wright A Hussein A Wymer J Howgate J Storey T. Evaluating digital maturity in public health systems: a statewide cross-sectional analysis of digital public health capacity in Missouri. Front Public Health. (2026) 13:1680904. doi: 10.3389/fpubh.2025.1680904, 41567781 PMC12816354

[82] Nyarko S Atinga RA Bardoe D Owusu AN. Digital health system integration in Ghana: a scoping review of governance arrangements, interoperability aspirations, and regulatory gaps. BMC Health Serv Res. (2026) 26:529. doi: 10.1186/s12913-026-14324-5, 41803867 PMC13085690

[83] Morley J Cowls J Taddeo M Floridi L. Ethical guidelines for COVID-19 tracing apps. Nature. (2020) 582:29–31. doi: 10.1038/d41586-020-01578-0, 32467596

[84] Kretzschmar ME Rozhnova G Bootsma MCJ van Boven M van de Wijgert JHHM Bonten MJM. Impact of delays on effectiveness of contact tracing strategies for COVID-19: a modelling study. Lancet Public Health. (2020) 5:e452–9. doi: 10.1016/S2468-2667(20)30157-2, 32682487 PMC7365652

[85] Francombe J Ali GC Gloinson ER Feijao C Morley KI Gunashekar S . Assessing the implementation of digital innovations in response to the COVID-19 pandemic to address key public health functions: scoping review of academic and nonacademic literature. JMIR Public Health Surveill. (2022) 8:e34605. doi: 10.2196/34605, 35605152 PMC9301563

[86] Donelle L Comer L Hiebert B Hall J Shelley JJ Smith MJ . Use of digital technologies for public health surveillance during the COVID-19 pandemic: a scoping review. Digit Health. (2023) 9:20552076231173220. doi: 10.1177/20552076231173220, 37214658 PMC10196539

[87] Ghildayal N Nagavedu K Wiltz JL Back S Boehmer TK Draper C . Public health surveillance in electronic health records: lessons from PCORnet. Prev Chronic Dis. (2024) 21:E51. doi: 10.5888/pcd21.230417, 38991533 PMC11262136

[88] Das S Kaur H Mukherjee S Chakraborty M Gupta R Roy S Developing a digital data platform for surveillance of food and water-borne pathogens in north East India: insight for public health advocacy. Front Public Health (2024);12:1422373. doi: 10.3389/fpubh.2024.1422373 PMC11381271 39253283

[89] Maaß L Zeeb H Rothgang H. International perspectives on measuring national digital public health system maturity through a multidisciplinary Delphi study. npj Digit Med. (2024) 7:92. doi: 10.1038/s41746-024-01078-9, 38609458 PMC11014962

[90] Wilson S Tolley C Mc Ardle R Lawson L Beswick E Hassan N . Recommendations to advance digital health equity: a systematic review of qualitative studies. npj Digit Med. (2024) 7:173. doi: 10.1038/s41746-024-01177-7, 38951666 PMC11217442

[91] Kirkcaldy RD Biggers B Bonney W Gordon J Yassine B Crawford B . Modernizing public health data systems and workforce capacity: the Centers for Disease Control and Prevention’s public health informatics fellowship program. J Public Health Manag Pract. (2025) 31:263–9. doi: 10.1097/PHH.0000000000002048, 39321350 PMC11757090

[92] Yao Y Cao J Tan Z Chen K. Developing a digital health competency assessment framework for public health services: a Delphi-AHP approach. Front Public Health. (2025) 13:1634261. doi: 10.3389/fpubh.2025.1634261, 40761928 PMC12319002

[93] Gorga A Barbati C Airapetian M Briganti GL Carnevali ED Cervellera F . Mapping digital public health training: are we preparing the European workforce? Front Public Health. (2026) 14:1778953. doi: 10.3389/fpubh.2026.1778953, 41756088 PMC12932469

[94] Maaß L Stark-Blomeier AL Tokgöz P Fischer F Schulz H Albrecht J. Building consensus on core teaching content of a digital public health curriculum: a Delphi study with public health experts in Germany. Front Public Health. (2026) 14:1799393. doi: 10.3389/fpubh.2026.1799393, 42404931 PMC13328418

[95] Iyamu I Wheelans C Haag D Roe I Chang HJ Donelle L . AI and algorithmic literacy among health workers: a scoping review through a digital health literacy lens. Front Public Health. (2026) 14:1802392. doi: 10.3389/fpubh.2026.1802392, 42110269 PMC13149243

[96] Attard C Silva AM Ahmed S Wong BLH Sultana R Albanese E . Designing interdisciplinary education in digital public health: a position paper. Front Public Health. (2026) 14:1866630. doi: 10.3389/fpubh.2026.1866630, 42454296 PMC13365167

[97] World Health Organization. Ethics and Governance of Artificial Intelligence for Health: WHO Guidance. Geneva: World Health Organization (2021).

[98] Wiens J Saria S Sendak M Ghassemi M Liu VX Doshi-Velez F . Do no harm: a roadmap for responsible machine learning for health care. Nat Med. (2019) 25:1337–40. doi: 10.1038/s41591-019-0548-6, 31427808

[99] Panch T Mattie H Atun R. Artificial intelligence and algorithmic bias: implications for health systems. J Glob Health. (2019) 9:020318. doi: 10.7189/jogh.09.020318, 31788229 PMC6875681

[100] World Health Organization. Regulatory Considerations on artificial Intelligence for Health. Geneva: World Health Organization (2023).

[101] Thirunavukarasu AJ Ting DSJ Elangovan K Gutierrez L Tan TF Ting DSW. Large language models in medicine. Nat Med. (2023) 29:1930–40. doi: 10.1038/s41591-023-02448-8, 37460753

[102] Lee P Bubeck S Petro J. Benefits, limits, and risks of GPT-4 as an AI chatbot for medicine. N Engl J Med. (2023) 388:1233–9. doi: 10.1056/NEJMsr2214184 36988602

[103] Harrer S. Attention is not all you need: the complicated case of ethically using large language models in healthcare and medicine. EBioMedicine. (2023) 90:104512. doi: 10.1016/j.ebiom.2023.104512, 36924620 PMC10025985

[104] Omiye JA Lester JC Spichak S Rotemberg V Daneshjou R. Large language models propagate race-based medicine. npj Digit Med. (2023) 6:195. doi: 10.1038/s41746-023-00939-z, 37864012 PMC10589311

[105] Singhal K Azizi S Tu T Mahdavi SS Wei J Chung HW . Large language models encode clinical knowledge. Nature. (2023) 620:172–80. doi: 10.1038/s41586-023-06291-2, 37438534 PMC10396962

[106] Berdahl CT Baker L Mann S Osoba O Girosi F. Strategies to improve the impact of artificial intelligence on health equity: scoping review. JMIR AI. (2023) 2:e42936. doi: 10.2196/42936, 38875587 PMC11041459

[107] U.S. Food and Drug Administration. Marketing Submission Recommendations for a Predetermined Change Control Plan for Artificial Intelligence-Enabled Device Software Functions: Guidance for Industry and Food and Drug Administration Staff. Silver Spring: U.S. Food and Drug Administration (2025).

[108] International Organization for Standardization International Electrotechnical Commission. ISO/IEC 42001:2023 Information Technology—Artificial Intelligence—Management system. Geneva: ISO (2023).

[109] International Organization for Standardization International Electrotechnical Commission. ISO/IEC 23894:2023 Information Technology—Artificial Intelligence—Guidance on risk Management. Geneva: ISO (2023).

[110] Moor M Banerjee O Shakeri Hossein Abad Z Krumholz HM Leskovec J Topol EJ . Foundation models for generalist medical artificial intelligence. Nature. (2023) 616:259–65. doi: 10.1038/s41586-023-05881-4, 37045921

[111] Reddy S. Generative AI in healthcare: an implementation science informed translational path on application, integration and governance. Implement Sci. (2024) 19:27. doi: 10.1186/s13012-024-01357-9, 38491544 PMC10941464

[112] Wagner JK Doerr M Schmit CD. AI governance: a challenge for public health. JMIR Public Health Surveill. (2024) 10:e58358. doi: 10.2196/58358, 39347615 PMC11457458

[113] Liao F Adelaine S Afshar M Patterson BW. Governance of clinical AI applications to facilitate safe and equitable deployment in a large health system: key elements and early successes. Front Digit Health. (2022) 4:931439. doi: 10.3389/fdgth.2022.931439, 36093386 PMC9448877

[114] Sendak M Balu S Hernandez AF. Proactive algorithm monitoring to ensure health equity. JAMA Netw Open. (2023) 6:e2345022. doi: 10.1001/jamanetworkopen.2023.45022, 38100115

[115] Chin MH Afsar-Manesh N Bierman AS Chang C Colón-Rodríguez CJ Dullabh P . Guiding principles to address the impact of algorithm bias on racial and ethnic disparities in health and health care. JAMA Netw Open. (2023) 6:e2345050. doi: 10.1001/jamanetworkopen.2023.45050, 38100101 PMC11181958

[116] World Health Organization. Ethics and Governance of Artificial Intelligence for Health: Guidance on Large Multi-Modal Models. Geneva: World Health Organization (2024).

[117] Tabassi E. Artificial Intelligence Risk Management Framework (AI RMF 1.0). NIST AI 100-1. Gaithersburg: National Institute of Standards and Technology (2023).

[118] Autio C Schwartz R Dunietz J Jain S Stanley M Tabassi E . Artificial Intelligence Risk Management Framework: Generative Artificial Intelligence Profile. NIST AI 600-1. Gaithersburg: National Institute of Standards and Technology (2024).

[119] European Parliament and Council. Regulation (EU) 2024/1689 of the European Parliament and of the Council of 13 June 2024 laying down harmonised rules on artificial intelligence and amending regulations (EC) No 300/2008, (EU) No 167/2013, (EU) No 168/2013, (EU) 2018/858, (EU) 2018/1139 and (EU) 2019/2144 and directives 2014/90/EU, (EU) 2016/797 and (EU) 2020/1828 (artificial intelligence act). Off J Eur Union. (2024) L 2024/1689. Avaliable online at http://data.europa.eu/eli/reg/2024/1689/oj

[120] Ramezani M Takian A Bakhtiari A Rabiee HR Ghazanfari S Sazgarnejad S. Research agenda for using artificial intelligence in health governance: interpretive scoping review and framework. BioData Min. (2023) 16:31. doi: 10.1186/s13040-023-00346-w, 37904172 PMC10617108

[121] Meskó B Topol EJ. The imperative for regulatory oversight of large language models (or generative AI) in healthcare. npj Digit Med. (2023) 6:120. doi: 10.1038/s41746-023-00873-0, 37414860 PMC10326069

[122] Cresswell K Rigby M Magrabi F Scott P Brender J Craven CK . The need to strengthen the evaluation of the impact of artificial intelligence-based decision support systems on healthcare provision. Health Policy. (2023) 136:104889. doi: 10.1016/j.healthpol.2023.104889 37579545

[123] Ueda D Kakinuma T Fujita S Kamagata K Fushimi Y Ito R . Fairness of artificial intelligence in healthcare: review and recommendations. Jpn J Radiol. (2024) 42:3–15. doi: 10.1007/s11604-023-01474-3, 37540463 PMC10764412

[124] Yang Y Lin M Zhao H Peng Y Huang F Lu Z. A survey of recent methods for addressing AI fairness and bias in biomedicine. J Biomed Inform. (2024) 154:104646. doi: 10.1016/j.jbi.2024.104646, 38677633 PMC11129918

[125] Mass General Brigham AI Governance Committee Saenz AD Centi A McCoy T Mantha AB Martin R . Establishing responsible use of AI guidelines: a comprehensive case study for healthcare institutions. npj Digit Med. (2024) 7:348. doi: 10.1038/s41746-024-01300-8, 39616269 PMC11608363

[126] Torabi F Squires E Orton C Heys S Ford D Lyons RA . A common framework for health data governance standards. Nat Med. (2024) 30:26–9. doi: 10.1038/s41591-023-02686-w 38191614

[127] Hu L Li D Liu H Chen X Gao Y Huang S . Enhancing fairness in AI-enabled medical systems with the attribute neutral framework. Nat Commun. (2024) 15:8767. doi: 10.1038/s41467-024-52930-1 PMC11464531 39384748

[128] Wells BJ Nguyen HM McWilliams A Pallini M Bovi A Kuzma A . A practical framework for appropriate implementation and review of artificial intelligence (FAIR-AI) in healthcare. npj Digit Med. (2025) 8:514. doi: 10.1038/s41746-025-01900-y, 40790350 PMC12340025

[129] Panteli D Adib K Buttigieg S Goiana-da-Silva F Ladewig K Azzopardi-Muscat N . Artificial intelligence in public health: promises, challenges, and an agenda for policy makers and public health institutions. Lancet Public Health. (2025) 10:e428–32. doi: 10.1016/S2468-2667(25)00036-2, 40031938 PMC12040707

[130] Brurberg KG Kjelle E Vardal J Sivanandan R. Artificial intelligence in emergency skeletal X-ray: post-deployment monitoring and clinical impact of incorrect AI results. Eur J Radiol. (2026) 199:112805. doi: 10.1016/j.ejrad.2026.112805, 41894992

[131] Lekadir K Frangi AF Porras AR Glocker B Cintas C Langlotz CP . FUTURE-AI: international consensus guideline for trustworthy and deployable artificial intelligence in healthcare. BMJ. (2025) 388:e081554. doi: 10.1136/bmj-2024-081554, 39909534 PMC11795397

[132] Collins GS Moons KGM Dhiman P Riley RD Beam AL van Calster B . TRIPOD+AI statement: updated guidance for reporting clinical prediction models that use regression or machine learning methods. BMJ. (2024) 385:e078378. doi: 10.1136/bmj-2023-078378, 38626948 PMC11019967

[133] Labkoff S Oladimeji B Kannry J Solomonides A Leftwich R Koski E . Toward a responsible future: recommendations for AI-enabled clinical decision support. J Am Med Inform Assoc. (2024) 31:2730–9. doi: 10.1093/jamia/ocae209, 39325508 PMC11491642

[134] Keyes T Callahan A Pandya AS Ambers N Banda JM Fuentes M . Why and how to monitor deployed AI systems in health care. NEJM Catal Innov Care Deliv. (2026) 7:CAT250372. doi: 10.1056/CAT.25.0372, 42418612

[135] Adnan HS Shidani A Clifton L Bankhead CR Perera-Salazar R. Implementation framework for AI deployment at scale in healthcare systems. iScience. (2025) 28:112406. doi: 10.1016/j.isci.2025.112406, 40384926 PMC12083986

[136] Owens K Griffen Z Damaraju L. Managing a “responsibility vacuum” in AI monitoring and governance in healthcare: a qualitative study. BMC Health Serv Res. (2025) 25:1217. doi: 10.1186/s12913-025-13388-z, 41023723 PMC12482494

[137] Crawford A Serhal E. Digital health equity and COVID-19: the innovation curve cannot reinforce the social gradient of health. J Med Internet Res. (2020) 22:e19361. doi: 10.2196/19361, 32452816 PMC7268667

[138] Veinot TC Mitchell H Ancker JS. Good intentions are not enough: how informatics interventions can worsen inequality. J Am Med Inform Assoc. (2018) 25:1080–8. doi: 10.1093/jamia/ocy052, 29788380 PMC7646885

[139] Sieck CJ Sheon A Ancker JS Castek J Callahan B Siefer A. Digital inclusion as a social determinant of health. npj Digit Med. (2021) 4:52. doi: 10.1038/s41746-021-00413-8, 33731887 PMC7969595

[140] van Kessel R Wong BLH Clemens T Brand H. Digital health literacy as a super determinant of health: more than simply the sum of its parts. Internet Interv. (2022) 27:100500. doi: 10.1016/j.invent.2022.100500, 35242586 PMC8861384

[141] Iyamu I Gómez-Ramírez O Xu AXT Chang HJ Watt S Mckee G . Challenges in the development of digital public health interventions and mapped solutions: findings from a scoping review. Digit Health. (2022) 8:20552076221102255. doi: 10.1177/20552076221102255, 35656283 PMC9152201

[142] Richardson S Lawrence K Schoenthaler AM Mann D. A framework for digital health equity. npj Digit Med. (2022) 5:119. doi: 10.1038/s41746-022-00663-0, 35982146 PMC9387425

[143] Alotaibi N Wilson CB Traynor M. Enhancing digital readiness and capability in healthcare: a systematic review of interventions, barriers, and facilitators. BMC Health Serv Res. (2025) 25:500. doi: 10.1186/s12913-025-12663-3, 40186200 PMC11969766

[144] Wallerstein N Duran B. Community-based participatory research contributions to intervention research: the intersection of science and practice to improve health equity. Am J Public Health. (2010) 100:S40–6. doi: 10.2105/AJPH.2009.184036, 20147663 PMC2837458

[145] Ocloo J Matthews R. From tokenism to empowerment: progressing patient and public involvement in healthcare improvement. BMJ Qual Saf. (2016) 25:626–32. doi: 10.1136/bmjqs-2015-004839, 26993640 PMC4975844

[146] Okwei ANN. Artificial intelligence and digital health equity: a post-pandemic evidence synthesis and implementation safeguards framework. Front Digit Health. (2026) 8:1785700. doi: 10.3389/fdgth.2026.1785700, 42231938 PMC13223092

